# Ipsilateral proximal and shaft femoral fractures treated with bridge-link type combined fixation system

**DOI:** 10.1186/s13018-020-01929-7

**Published:** 2020-09-10

**Authors:** Liangqi Kang, Hui Liu, Zhenqi Ding, Yiqiang Ding, Wei Hu, Jin Wu

**Affiliations:** 1 Department of Orthopedics, The Affiliated Southeast Hospital of Xiamen University, Zhangzhou, 363000 China; 2 The Affiliated Southeast Hospital of Xiamen University, Zhangzhou, 363000 China

**Keywords:** Femoral shaft fractures, Femoral proximal fractures, Bridge-link type combined fixation system (BCFS), MIPPO

## Abstract

**Background:**

Although many treatments for ipsilateral proximal and shaft femoral fractures have been developed, controversy exists regarding their optimal management. The purpose of this retrospective study was to discuss the effectiveness of the bridge-link type combined fixation system (BCFS) and evaluate functional outcomes in treating patients with these complex fractures.

**Patients and methods:**

We retrospectively reviewed 14 cases of ipsilateral proximal and shaft femoral fractures treated from January 2012 to December 2016. All cases were treated by BCFS combined with minimally invasive percutaneous plate osteosynthesis (MIPPO). Clinical and radiographic data were collected during regular post-operative follow-up visits. Functional outcomes were determined according to the Friedman and Wyman scoring system.

**Results:**

The proximal femoral fractures were emergency diagnoses in 11 cases and delayed diagnoses in 3 cases. The delay time was 5–6 days, with an average of 5.3 days. The mean operation time was 179.6 min (range 135–231 min) with a blood loss volume that ranged from 430 to 535 ml (average 483.6 ml). Follow-up was conducted in 13 cases between 9 and 30 months post-operation, with an average follow-up time of 17.3 months. The proximal femoral fractures were united in 12 cases at the final follow-up. One case had nonunion 13 months after the operation, underwent valgus intertrochanteric osteotomy, and healed 6 months later. The femoral shaft fractures obtained rigid union at the latest follow-up in 12 cases. One case endured nonunion 12 months after the operation. After the revision surgery and iliac bone grafting, the fracture healed 6 months later. Eight of the cases had good functional results, 4 had fair results, and results were poor in 1 case at the final follow-up.

**Conclusions:**

The treatment of ipsilateral proximal femoral and shaft fractures with BCFS in combination with MIPPO demonstrated a high likelihood of union for both fractures and good functional results.

## Introduction

The first case report of an ipsilateral proximal and shaft femoral fracture was published in 1953 [1]. Since then, orthopedists have paid more attention to this combined injury pattern and provided recommendations regarding its diagnosis and treatment. Ipsilateral proximal and shaft femoral fracture presentation is rare, accounting for approximately 2.5–6% of femoral fractures [2]. The average patient age is 35 years, and 75% of patients are male [3]. Most cases are caused by high-impact injuries, such as motor vehicle accidents or falls, and are often associated with multisystem (head, chest, or abdominal) associated injuries [3, 4]. Twenty to 40% of patients also have ipsilateral knee injuries, including ligamentous injury, tibial plateau fracture, patellar fracture, or knee dislocation [4]. With the increase in high-energy injuries in recent years, the ipsilateral proximal and shaft femoral fracture morbidity rate is rising.

Attributed injury mechanisms include an axial force transmitted proximally on a flexed femur toward the acetabular roof, causing the combination of femoral shaft fracture and ipsilateral proximal femoral fracture [2–5]. Often, diagnosis of proximal femoral fracture is delayed in 19–31% of patients because of the high Injury Severity Score (ISS), other injuries associated with shaft femoral fracture, and unapparent clinical signs of the hip [6]. Surgical stabilization with implants for ipsilateral proximal and shaft femoral fractures is recommended by most published reports, and various techniques and implants have been developed to manage such fractures [2, 3, 5–9]. However, optimal treatment of these complex fractures is still debated, with different techniques and implants each having their own advantages and disadvantages. Further, the incidence of femoral fracture nonunion is 2–10%, and the rate of femoral neck postoperative complications could be as high as 25% [2, 4, 10–12].

Based on the principles of biological osteosynthesis which emphasize both the stability of the fracture region and maintenance of soft tissue vitality, a clamp rod internal fixator (CRIF) system was designed by the AO Development Institute [13]. However, due to the lack of locking structure between plate and screws, there is poor bone-holding power; thus, CRIF is not the preferred implant for osteosynthesis of long bones [14, 15]. BCFS (Walkman Biomaterial Co., Ltd., Tianjin, China, patent number: ZL 2005 2 0022267.7) is a novel clamp rod internal fixation device that has been widely used in the fixation of fractures of the upper and lower limbs or pelvis. Biomechanical and clinical analysis showed favorable results with BCFS for long bone fractures [16]. To our knowledge, there are few reports regarding the treatment of ipsilateral proximal and shaft femoral fractures with BCFS, and this retrospective study was performed to evaluate BCFS for the treatment of ipsilateral proximal and shaft femoral fractures.

## Patients and methods

### Clinical data

This study was a retrospective analysis of existing clinical cases and approved by the institutional review board. Fourteen patients (10 males and 4 females) were enrolled in the study between January 2012 and December 2016. Detailed clinical patient parameters are shown in Table 1. The average age at enrolment was 35.4 years (range: 17–60 years). Twelve patients suffered from closed fractures, and 2 had open fractures (all Gustilo I). The causes of injury included traffic accidents (10 patients) and falls from a significant height (4 patients). Two patients had fractures associated with hematopneumothorax, one with ipsilateral tibial fracture, one with liver contusion, one with traumatic brain injury, one with contralateral tibial fracture, and one with ipsilateral patella fracture. There were twelve neck fractures, one trochanteric, and one subtrochanteric fractures. According to the AO-OTA classification, there were three type A, three type B, and eight type C femoral shaft fractures. According to Garden’s classification, two femoral neck fractures were type I, seven were type II, two were type III, and one was type IV. Garden type I and II fractures are considered nondisplaced, and Garden types III and IV are considered displaced [17]. Anatomically, one of the neck fractures was subcapital fracture, ten were basicervical fractures, and one was transcervical fractures. According to Evans-Jensen and Seinsheimer classification, there was one type II trochanteric fracture and one type II subtrochanteric fracture, respectively. All patients were diagnosed by clinical symptomatology, X-ray, and computer tomography (CT).

**Table 1:** Clinical parameters of the patients

No	Gender/age (years)	Injury mechanism	Femoral shaft fracture (Gustilo/AO-OTA type)	Proximal femoral fracture (fracture type/anatomical location)	Complicated injury
1	Male/29	Traffic accident	Closed/C	Garden I/Basicervical fracture	–
2	Male/42	Traffic accident	Open I/C	Garden II/Basicervical fracture	–
3	Male/27	Fall from height	Closed/A	Evans-Jensen II/Trochanteric fracture	Hematopneumothorax
4	Male/36	Traffic accident	Closed/C	Garden II/Basicervical fracture	–
5	Male/33	Traffic accident	Closed/B	Garden III/Basicervical fracture	Ipsilateral tibial fracture
6	Male/22	Fall from height	Closed/C	Garden II/Transcervical fracture	Liver contusion
7	Male/40	Traffic accident	Closed/A	Garden II/Basicervical fracture	–
8	Female/45	Fall from height	Closed/C	Garden IV/Basicervical fracture	Traumatic brain injury
9	Male/30	Traffic accident	Closed/C	Garden II/Basicervical fracture	–
10	Female/36	Traffic accident	Closed/C	Garden II/Basicervical fracture	Ipsilateral patella fracture
11	Male/30	Traffic accident	Closed/C	Garden III/Subcapital fracture	Contralateral tibial fracture
12	Male/60	Traffic accident	Open I/A	Seinsheimer II/Subtrochanteric fracture	–
13	Female/48	Traffic acciden	Closed/B	Garden I/Basicervical fracture	Hematopneumothorax
14	Female/17	Fall from height	Closed/B	Garden II/Basicervical fracture	–

### Pre-operative preparation

Proximal tibial skeletal traction was performed immediately after patients with closed fractures were admitted to the hospital (calcaneal traction was performed in patients accompanied with patella or tibial fractures). Patients with Gustilo I open fractures first underwent debridement and suturing, after which they received proximal tibial skeletal traction. All patients with open fractures received intravenous antibiotics for 24 h postoperatively. X-ray and CT examinations were used to visualize fracture displacement for the operative plan. As soon as their condition was stabilized, all patients underwent surgical treatment.

### Surgical procedure

The patient was placed in the supine position and was under either general or epidural anesthesia. For femoral neck fractures, temporary fixation was performed with Kirschner wires after the fractures were reduced with appropriate abduction and internal rotation. For femoral trochanteric or subtrochanteric fractures, fixation was performed with a reconstruction locking plate or cortical screws after satisfactory reduction. Then, a proximal femoral anatomical clamp was placed below the apex of the greater trochanter, and, after insertion of the guide pins, 3 proximal cannulated screws were placed into the head. For femoral shaft fractures, limited open reduction was performed. After elevating the vastus lateralis muscle from the intermuscular septum, the lateral cortex of the femoral shaft was visualized. The affected limb was pulled longitudinally, and a periosteum elevator was inserted between the fracture site, manipulated to elevate the fragments. One 6–8 hole reconstruction locking plate was placed at the anterior site of the femur, and the distal and proximal ends of the fracture were each fixed with 3 unicortical locking screws. Finally, the lateral femoral condyle was exposed through a limited open approach. A distal femoral anatomical clamp was then placed on the lateral femoral condyle, and the length of the connection rods used between the proximal and distal femoral anatomical clamps was measured. Two connection rods with appropriate length were taken, three or four sliding clamps were installed on the connection rods, and then the connection rods were inserted from the distal femur into the proximal femur through the soft tissue tunnel. The distal and proximal ends of the connection rods were put into the anatomical clamps, and the position of each sliding clamp was adjusted. At last, the set screws were screwed into the clamps to tighten the connection rod and hold the clamp tightly. Reduction and femoral alignment was confirmed by fluoroscopy. Finally, the wound was irrigated and closed, and a drain was placed.

### Postoperative management

The postoperative ambulatory program consisted of non-weight-bearing activities for 8–12 weeks and then gradual partial weight-bearing activities. Full-weight bearing on the affected limb was commenced once the presence of radiological consolidation was noted.

### Outcome assessment

Outcome assessment after surgery was evaluated according to Friedman and Wyman scoring system [18]. A “good” result meant no limitation of activities of daily living, no pain, and loss of less than 20% of hip or knee motion. A “fair” result indicated mild limitation of activities of daily living, mild to moderate pain, and a loss of motion of 20–50%. A “poor” result indicated moderate limitation of activities of daily living, severe pain, and loss of motion of more than 50%. Radiological parameters included X-ray and/or CT scans were taken every 1 month post-operation to evaluate the bony fusion until they showed solid continuous callus formation. Based on previous studies [9], femoral neck fractures were considered united if anteroposterior (AP) and lateral radiographs showed that three of four cortices had trabeculae bridging the fracture site. When the patient could tolerate full-weight bearing without pain or radiological consolidation of the fracture was visible, the femoral shaft fracture was considered healed. If complete radiological consolidation of the fracture was not present by 6 months postoperatively, it was classified as a delayed union. When consolidation was not present by 12 months, the fracture was classified as nonunion [19].

## Results

**Fig. 1:**
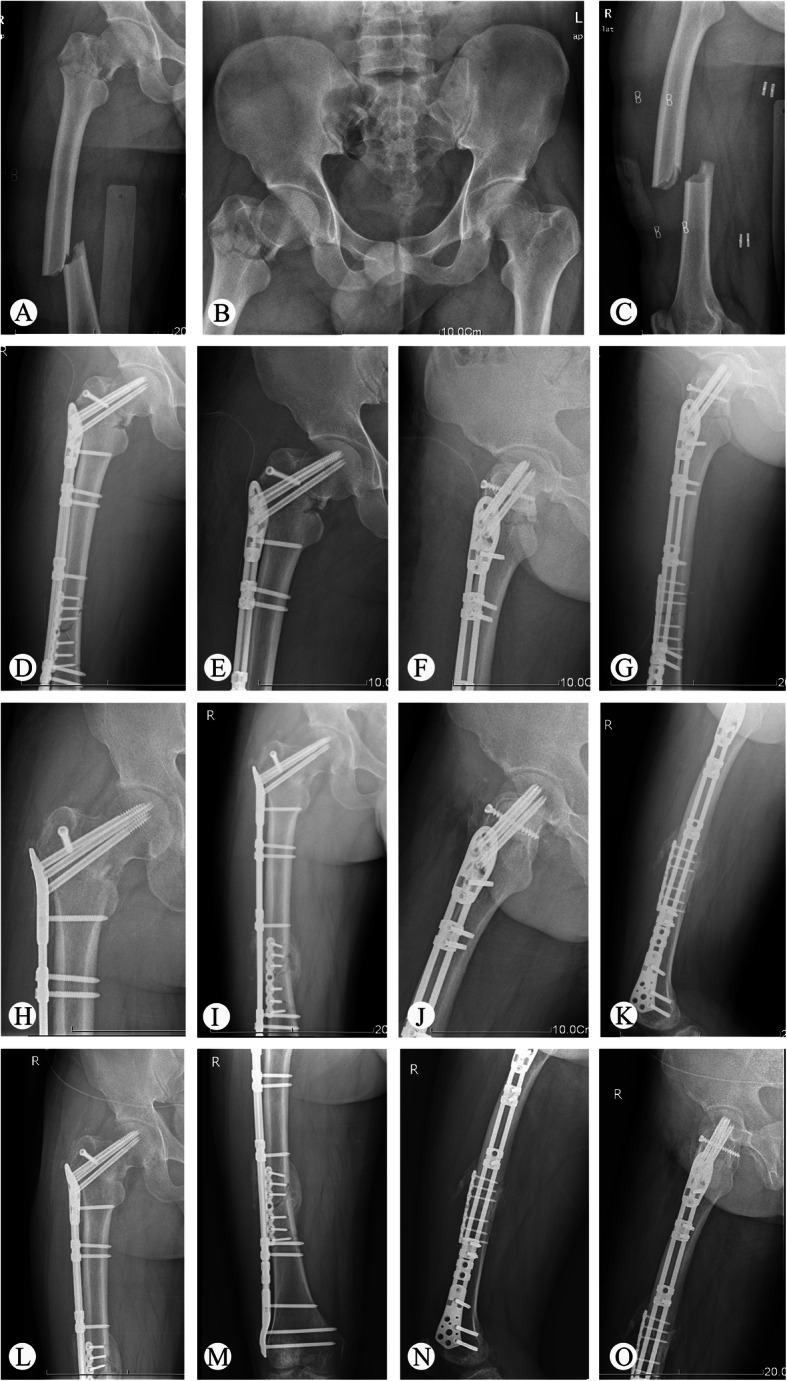
Representative images of patient 3. **a**–**c** X-ray at admission. **d**–**g** X-ray at 7 days after the operation. **h**–**k** X-ray at 4 months follow-up. **l**–**o** X-ray at last follow-up

**Fig. 2:**
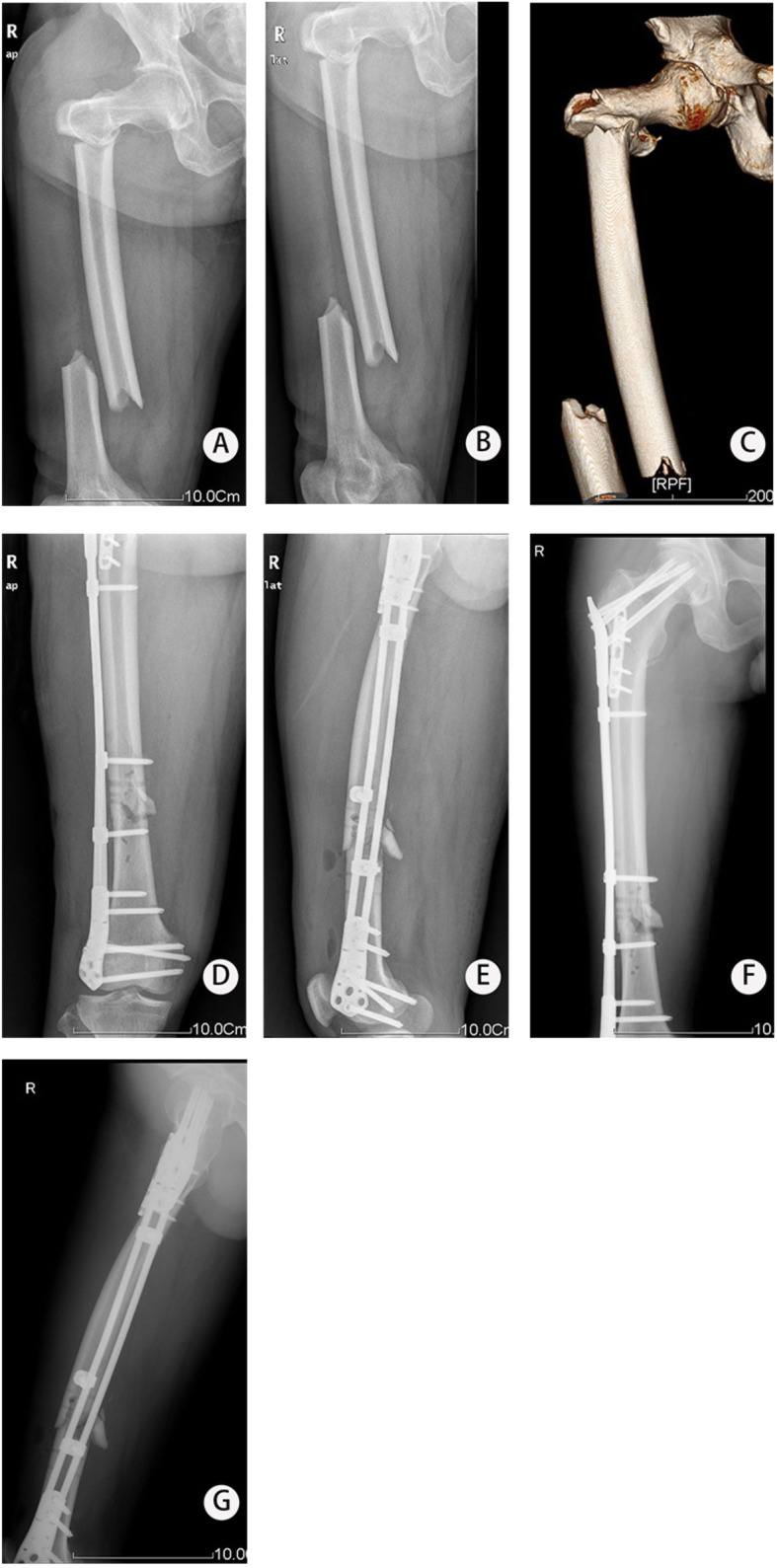
Representative images of patient 12 who lost connection at 3-month follow-up. **a**–**c** X-ray at admission. **d**–**i** X-ray at 3 days after operation

The mean operation time was 179.6 min (range 135–231 min) with a blood loss volume ranged from 430 to 535 ml (average 483.6 ml). Follow-up was conducted in thirteen patients between 9 and 30 months post-operation, with an average follow-up time of 17.3 months. One patient lost connection at 3-month follow-up. The proximal femoral fractures were united in 12 cases at the final follow-up. The duration for bone union ranged from 3 to 6 months, with an average of 4.2 months. One case had nonunion 13 months after the operation and underwent valgus intertrochanteric osteotomy and healed 6 months later. The femoral shaft fractures obtained rigid union in 12 cases at the latest follow-up. The duration for bone union ranged from 3 to 7 months, with an average of 5.0 months. One case endured nonunion 12 months after the operation. After the revision surgery and iliac bone grafting, the fracture healed 6 months later. According to Friedman and Wyman scoring system, 8 of the cases had a good functional result, in 4 the result was fair, and in 1, it was poor at the final follow-up. The complications included infection in 1, varus angulation of femoral neck in 1, nonunion of femoral neck fracture in 1, nonunion of femoral shaft fracture in 1, and avascular necrosis of femoral head in 1 (Table 2). Typical cases are shown in Fig. 1 (patient 3) and Fig. 2 (patient 12).

**Table 2:** Clinical parameters of the patients

No	Delayed diagnosis (days)	Time to surgery (days)	Operation time (mins)	Blood loss (ml)	Follow-up (months)	Neck union (months)	Shaft union (months)	Outcomes	**Complications**
1	–	3	148	460	18	3	5	Good	–
2	5	11	162	510	22	4	4	Good	Superficial infection
3	–	7	206	565	30	6	6	Good	–
4	–	4	155	455	17	4	17	Fair	Femoral shaft nonunion
5	–	8	197	525	12	5	4	Fair	Femoral neck varus angulation
6	6	14	183	500	25	4	5	Good	–
7	–	2	174	450	9	3	3	Good	–
8	–	11	191	495	21	5	6	Good	–
9	–	3	178	445	14	4	5	Good	–
10	–	3	194	510	12	3	7	Fair	–
11	–	6	231	535	16	5	6	Poor	Osteonecrosis of the femoral
12	–	20	166	520	Lost	–	–	–	head
13	5	9	157	430	19	19	4	Fair	–
14	–	3	135	455	11	4	5	Good	Femoral neck nonunion

## Discussion

Although ipsilateral proximal and shaft femoral fractures are not common, they present a diagnostic and therapeutic challenge for orthopedic surgeons. The majority of the patients in the reported case series were young males with high-energy trauma. Due to the frequency with which the injury is seen in vehicle crash front seat drivers and passengers, the typical damage mechanism has been postulated to be the result of a longitudinal compression force on a flexed and abducted hip. The femoral shaft fracture is typically comminuted, located in the middle third of the diaphysis, and open in 15–33% of cases. The femoral neck fracture is usually basicervical, vertically oriented, and nondisplaced in 60% of cases [20].

Diagnosis of proximal femoral fracture is easily delayed or missed. The incidence of delayed diagnosis is around 19–31%, and the incidence of missed diagnosis may be 13–31% [6]. Early diagnosis is generally difficult because the fracture is most often basilar and the symptoms are mild in the early phase of the disease. Furthermore, surgeons pay more attention to the often life-threatening associated injuries (head, chest, or abdominal) [4]. Therefore, patients with femoral shaft fractures should undergo thorough radiography of the pelvis and both hips as a standard protocol. Additionally, CT scan examination should be performed if necessary. However, a nondedicated CT scan does not always reveal the femoral neck fracture preoperatively [21]. Objective sound waves can also be used as a diagnostic test or screening tool in the assessment of occult hip fractures [22]. Moreover, a recent study found that rapid limited-sequence pelvis MRI for patients with femoral shaft fractures could identify femoral neck fractures that were not diagnosed on thin-cut high-resolution CT [23]. In our present series, nine of the patients were young males (64.2%), two had open femoral shaft fractures (14.3%), seven had multisystem injuries (50.0%), and two had ipsilateral knee injuries (14.3%). Further, nine of the neck fractures were nondisplaced (75.0%), one was a subcapital fracture (8.3%), ten were basicervical fractures (83.3%), and one was a transcervical fracture (8.3%). Delayed diagnosis was observed in 3 cases (21.4%). The average delay time was 5.3 d (range, 5–6 days), and all of these cases were transferred from other departments to orthopedics. All of the results in our study were consistent with previous reports.

Non-operative treatment of either the proximal femoral fracture or shaft fracture is generally avoided except in extenuating circumstances, and poor results have been reported [24]. A series of surgical treatment strategies have been described for this combined injury. Orthopedic surgeons need to consider three issues for an optimal preoperative plan: (1) appropriate timing of surgical fixation, (2) which fracture to stabilize first, and (3) the optimal internal fixation for these complex fractures. The purpose of ipsilateral proximal and shaft femoral fracture surgical management is to achieve the anatomical reduction of the proximal femoral fracture and rigid fixation of both fractures. Studies have recommended that the operation be performed within 8 h, 12 h, 24 h, 72 h, or 1 week [9, 25–27]. It is generally accepted that early surgical treatment can reduce patient complications and morbidity and allow for earlier functional exercise and rehabilitation. Therefore, after the patient’s general condition has stabilized, early reduction and fixation of this combined injury pattern are necessary. There is no consensus as to which fracture should be managed first. Some recommend that the femoral neck fracture should be always provisionally fixed first, either by closed or open methods, to avoid displacement of a nondisplaced or minimally displaced fracture and to ensure anatomic reduction and optimal stabilization of the neck, preventing osteonecrosis and nonunion [8]. Others advocate for fixation of the femoral shaft first to allow better control of the leg during the more technically challenging femoral neck reduction [28]. Generally speaking, we favored that the fixation should depend on the femoral neck fracture pattern. This therapeutic strategy is satisfactory with nondisplaced neck fractures, as neck fractures could be fixed in situ and further displacement is prevented. Otherwise, the femoral shaft should be fixed first to allow for better control of the leg during the reduction of the displaced femoral neck fracture.

Various constructs and surgical techniques have been described for the treatment of ipsilateral proximal and shaft femoral fractures, including single constructs or dual constructs for both fractures [4, 6, 7, 25, 29, 30]. Single constructs include reconstruction intramedullary nails with interlocked screw fixation for the neck fracture via the nail, long proximal femoral nails (PFN-long) with anti-rotation, long sliding hip screws, and long proximal femoral locking plates [7, 9, 20]. Some favor the reconstruction nail, which allows possible closed reduction and biomechanical fixation with minimal incision, achieving biological fixation of both fractures and reduced intraoperative blood loss. However, this advantage may be outweighed by the high technical demand of accurately placing the proximal screws into the head and neck [7]. Additionally, reconstruction nailing should not be performed in displaced femoral neck fractures because it is difficult to achieve reduction [7]. PFN-long could stabilize and fix both fractures simultaneously, as described by Wang et al. [9]. Biomechanical studies have also shown that the helical blade could provide higher stability than the lag screw [31]. However, the availability of only three nail lengths and one diameter presents certain drawbacks [9].

Dual constructs include (1) retrograde nailing combined with dynamic hip screw (DHS) or lag screws [6]; (2) a dynamic compression plate (DCP) with DHS, cannulated cancellous screws, or a proximal femoral locking plate [2]; and (3) antegrade femoral intramedullary nails with cannulated cancellous screws [27]. When compared with single constructs, dual constructs have many advantages, such as easier immobilization and superior biomechanical fixation [7, 8]. However, their clinical application is limited by complications related to the knee, risk of fracture nonunion (particularly for retrograde nailing combined with DHS or lag screws), greater intraoperative trauma, and higher implant cost [28].

The rate of avascular necrosis of the femoral head in ipsilateral proximal and shaft femoral fractures is lower than that seen with isolated femoral neck fractures. In these fractures, the reported incidence in various series has ranged from 1.2 to 5% [5]. Nonunion of the femoral neck can also occur. In a series of 95 patients treated with femoral neck screw fixation and retrograde reamed intramedullary nailing, the nonunion rate of the femoral neck was only 2% [32]. Complications related to ipsilateral femoral shaft fractures are more common than for the neck in almost all published series. Compared to isolated femoral shaft fractures, associated shaft fractures have a higher average time to the union (20.3–27.3 weeks) and higher rates of nonunion 0–23% and malunion 3.7–40% [33, 34]. This may be due to a difference in treatment, an inherent injury characteristic, a result of post-operative protocol changes or a combination of these factors [24]. In our present series, the rate of avascular necrosis and nonunion of the femoral head was 8.3% (1/12), respectively. The nonunion of femoral shaft fracture was found in one case (7.7%). All of the results were in accord with previous reports.

**Fig. 3:**
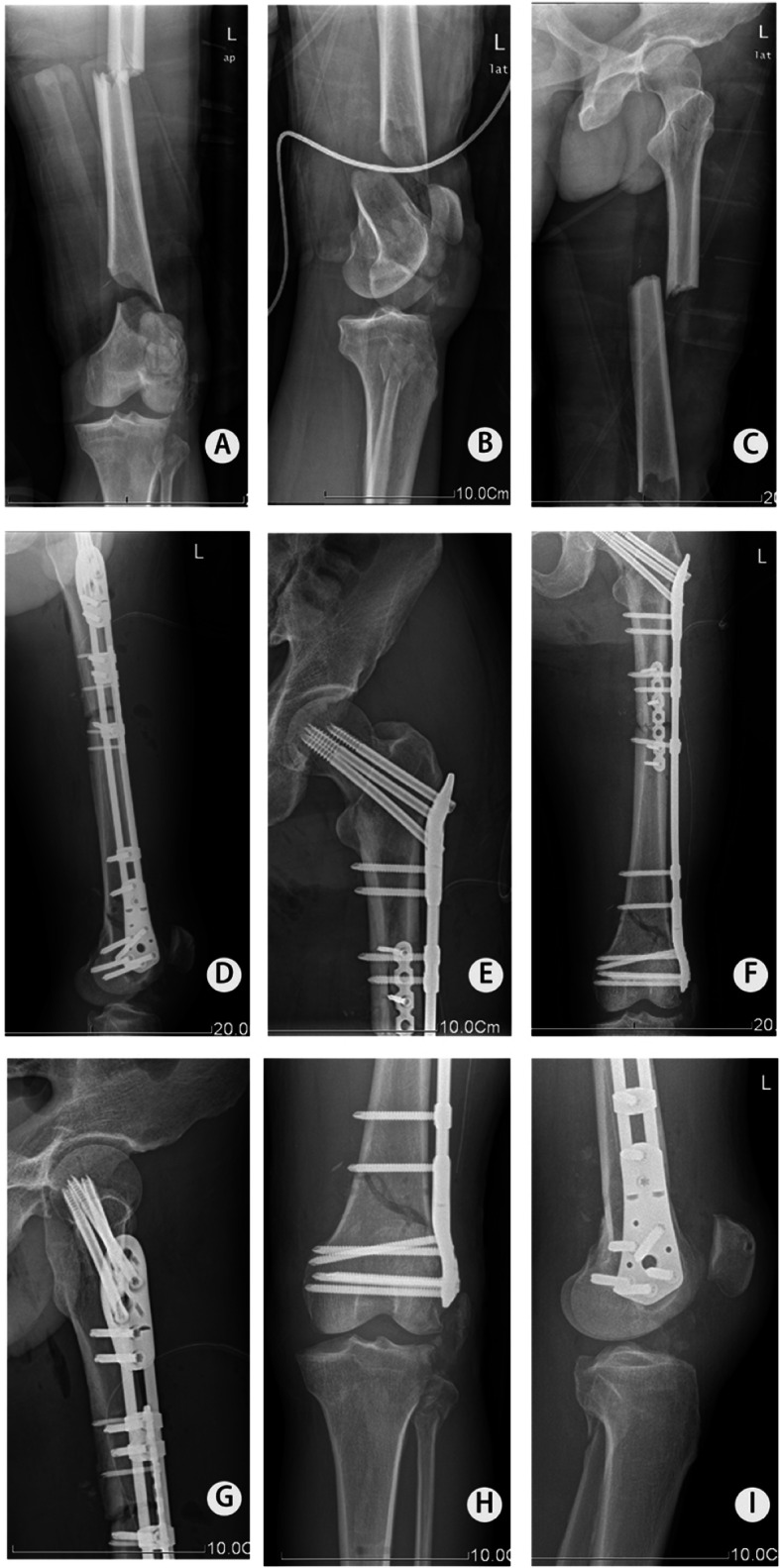
A 63-year-old male who suffered a motor vehicle accident and dignosed as ipsilateral femoral neck, shaft, and distal fracture. **a**–**c** X-ray at admission. **d**–**i** X-ray at 5 days after the operation

In order to minimize complications and trauma, all cases in our study were treated by BCFS in combination with MIPPO. The BCFS was comprised of clamps, connection rods, screws (set screw, locking screw, and cortical screw), and other accessory instruments (Supplemental material). Based on the patient needs, the surgeon chose different sizes of clamps and connection rods during the operation. When the set screw was screwed into the clamp, its nut tightened the connection rod and held the clamp tightly. Thus, the clamps, screws, and connection rods were locked as a whole to support firm and stable fixation of both the proximal and shaft femoral fractures. Compared to other treatment methods, the BCFS has relatively easy application and less trauma. Four different treatment methods (antegrade reamed intramedullary nailing and cancellous screw, DHS and low-contact dynamic compression plate (LCDCP), cancellous screw and LCDCP, and reconstruction nailing) were used for 43 patients with ipsilateral femoral neck and shaft fractures; these procedures had a mean surgery duration of 280 min and resulted in a mean blood loss of 428.6 ml [35]. The BCFS takes 179.6 min operation time with an average blood loss volume of 483.6 ml, reflecting the relative ease of manipulation. Furthermore, the curative effects, including fracture healing, complications, and postoperative functional indexes, are basically consistent with previous literature [35]. Moreover, BCFS has a wide scope of application, even in the treatment of ipsilateral fractures of the femoral neck, shaft, and distal end cases (Fig. 3).

## Conclusion

Here, we have presented our results from treating concomitant ipsilateral fractures of the proximal femur and femoral shaft by BCFS. Our summarized clinical effects show that BCFS is a good option for the treatment of complex fractures with biological fixation of both fractures and satisfactory results. However, future studies featuring longer outcome measures and a higher number of patients are still needed.

## Supplementary information

**Additional file 1.**

## Abbreviations

AP: Anteroposterior
BCFS: Bridge-link type combined fixation system
CRIF: Clamp rod internal fixator
CT: Computer tomography
DCP: Dynamic compression plate
DHS: Dynamic hip screw
ISS: Injury Severity Score
LCDCP: Low-contact dynamic compression plate
MIPPO: Minimally invasive percutaneous plate osteosynthesis
