# Supplementary figures and images for: Ipsilateral proximal and shaft femoral fractures treated with bridge-link type combined fixation system
Source: J Orthop Surg Res. 2020 Sep 10;15:399. doi: 10.1186/s13018-020-01929-7 (PMC7488305; doi:10.1186/s13018-020-01929-7)

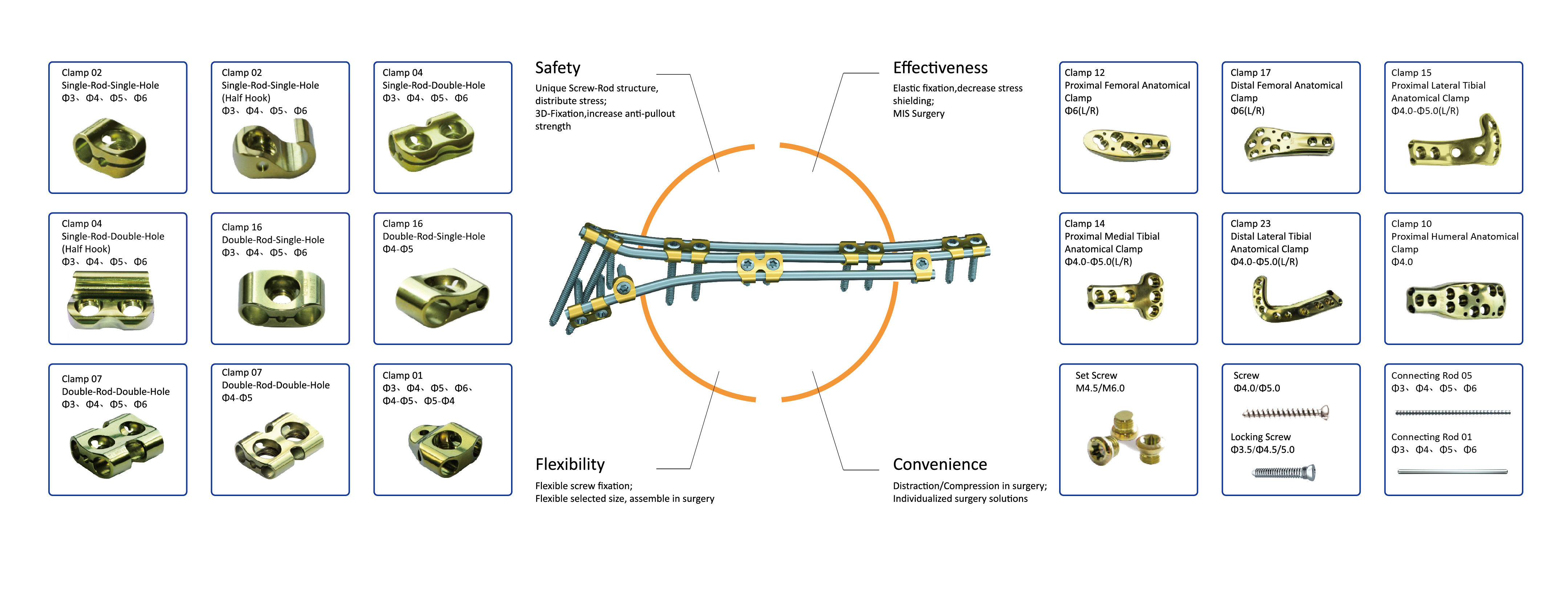

Supplement: Supplementary file 1 — Additional file 1. [file 13018_2020_1929_MOESM1_ESM.jpg]
